# Pesticides With Potential Thyroid Hormone-Disrupting Effects: A Review of Recent Data

**DOI:** 10.3389/fendo.2019.00743

**Published:** 2019-12-09

**Authors:** Michelle Leemans, Stephan Couderq, Barbara Demeneix, Jean-Baptiste Fini

**Affiliations:** Muséum National d'Histoire Naturelle, CNRS UMR 7221, Laboratoire Physiologie moléculaire de l'adaptation, Paris, France

**Keywords:** thyroid hormones, pesticides, endocrine disruptors, organochlorine, organophosphates, pyrethroids, neonicotinoids, neurodevelopment

## Abstract

Plant Protection Products, more commonly referred to as pesticides and biocides, are used to control a wide range of yield-reducing pests including insects, fungi, nematodes, and weeds. Concern has been raised that some pesticides may act as endocrine disrupting chemicals (EDCs) with the potential to interfere with the hormone systems of non-target invertebrates and vertebrates, including humans. EDCs act at low doses and particularly vulnerable periods of exposure include pre- and perinatal development. Of critical concern is the number of pesticides with the potential to interfere with the developing nervous system and brain, notably with thyroid hormone signaling. Across vertebrates, thyroid hormone orchestrates metamorphosis, brain development, and metabolism. Pesticide action on thyroid homeostasis can involve interference with TH production and its control, displacement from distributor proteins and liver metabolism. Here we focused on thyroid endpoints for each of the different classes of pesticides reviewing epidemiological and experimental studies carried out both in *in vivo* and *in vitro*. We conclude first, that many pesticides were placed on the market with insufficient testing, other than acute or chronic toxicity, and second, that thyroid-specific endpoints for neurodevelopmental effects and mixture assessment are largely absent from regulatory directives.

## Background

Plant Protection Products (PPPs) (herein referred to as pesticides) are used to control noxious pests and disease causing organisms including insects, fungi and unwanted plants (1) (EU Commission^1^). A major shift from inorganic pesticides, such as lead arsenate, to synthetic organic chemicals occurred in the late 1940s. These approaches revolutionized pest control efficiency to such a degree that synthetic pesticides were rapidly integrated into the ongoing industrialization of agriculture, public health programs and use by individuals. The revolution in industrial farming increased crop production and quality, with ensuing global production of synthetic pesticides escalating at a yearly rate of 10% from the 1950s, reaching upwards of 3 million tons by the turn of the century and 4 million tons in 2016 (2–4). Along with increasing production volumes, numerous synthetic pesticides appeared on the market, often due to development of acquired resistance by target species and/or regulatory restrictions brought by health or environmental concerns. The first synthetic pesticides introduced in the 1940s were the **organochlorine pesticides (OCP**), followed by **organophosphates (OP)** in the 1960s and **carbamates** in the 1970s. **Pyrethroid** production began in the 1980s, with the more recent compounds such as **neonicotinoids** and **phenylpyrazoles** being added in the 1990s (5).

Despite the early warnings of Rachel Carson in 1962, the intensity of pesticide use placed an increasing burden on the environment with limited consideration for the full extent of consequences. Acute or chronic exposure to a variety of pesticides occurring through cutaneous, respiratory or dietary routes are supported by current human biomonitoring efforts as well as studies on wild-life (6, 7). Simultaneously, a substantial body of evidence confirming the adverse health effects and ecological impact of PPP (including biocides) has been accumulating since the 1960s when pesticides, once heralded as a “magic bullet,” made usage more controversial.

Carson's premonition of the impact of the organochlorine insecticide DDT^2^ on eggshell thinning in birds was later shown to be caused by endocrine-mediated effects, specifically, the estrogenic action of DDT's main metabolite p,p′-DDE^3^ (8, 9). The term “endocrine disruptor” gained traction in the early 90s when used to describe the interference of several man-made chemicals, including certain pesticides, on estrogen, androgen, thyroid and steroid pathways (10, 11). While the acute toxic effects of pesticides on target, non-target species or occupational workers are quite well-documented (12–14), the effects of low doses relevant to concentrations assessed in foodstuffs and the environment are relatively neglected. Traditionally, regulatory systems rely on dose-response models and determine a threshold for safe levels of exposure, but potentially overlook the presence of non-monotonic dose-response curves below the toxicological no-observed-adverse-effect level (NOAEL) (15). This issue is particularly important given that many pesticides act as endocrine disrupting chemicals (EDCs), capable of interfering with natural hormones even at low doses and hence affect the normal development and function of multiple organs (16–19). Additionally, EDCs may exert specific effects during sensitive time-windows of development with adverse health outcomes occurring later in life—bringing the matter of timing of exposure during vulnerable periods, such as prenatal or postnatal life, increasingly into focus in both regulatory and fundamental research (20, 21).

## Overview of the Hypothalamus-Pituitary-Thyroid Axis

During the last 30 years insights into the role of thyroid hormones (TH) at different levels of biological organization have contributed to a better physiological understanding of their function. In humans, TH is essential for the development of the brain, inner ear, eye, heart, kidneys, bone and skeletal muscle, amongst other tissues (22), but also for fine regulation of energy metabolism (23). The essential role of TH during vertebrate development has been amply reviewed (24–26), however neurodevelopment warrants special attention. Strikingly, THs are crucial for normal brain development which is dramatically illustrated by cretinism, a syndrome induced by a severe lack of TH or iodine during embryo-fetal and post-natal development (27). In addition, disruption at any of the multiple levels along the hypothalamus-pituitary-thyroid axis (HPT-axis) axis during this vulnerable period of development, particularly early pregnancy (28, 29) can lead to deleterious effects on offspring IQ. These effects can be exerted through modifications of TH levels in the blood stream and within specific tissues, with subsequent modulation of TH-dependent actions in the nervous system (transcription, proliferation, neurogenesis, gliogenesis, migration), resulting potentially in altered brain structure and behavior (19, 28)^4^.

To summarize TH production and physiology very succinctly, TH synthesis in the thyroid colloid requires iodine, which circulates as iodide ion. In the thyroid gland, iodide is combined with the amino acid tyrosine to produce thyroxine (T4) or triiodothyronine (T3). Synthesis of THs (T4 and T3) is tightly controlled by the HPT-axis. The hypothalamus produces thyrotropin-releasing hormone (TRH), which triggers production of thyroid stimulating hormone (TSH) by the anterior pituitary. TSH is released into the bloodstream and binds to receptors on thyroid follicular cells of the thyroid gland. TSH stimulates iodide uptake mediated by the sodium/iodide symporter (NIS). After oxidation of iodide by thyroperoxidase (TPO), organification of iodide which consists of incorporation of iodide into thyroglobulin (Tg) is required to produce precursors of T3 and T4 (30). THs exert negative feedback on their upstream regulators thereby controlling hormone levels. Increases in T3 or T4 inhibit production of TSH and TRH by the pituitary and hypothalamus, respectively. Inversely, as T3 and T4 decrease, TSH and TRH genes are activated (31). In the bloodstream, THs are almost entirely bound to serum distributing proteins, such as transthyretin (TTR), albumin or thyroxin-binding globulin (TBG). Less than 0.001% of total T4 and T3 are available as free T4 or T3 (FT4 or FT3) and can enter cells through trans-membrane-transporters. Several transporters carry THs, including monocarboxylate transporters (MCTs), several members of the organic anion-transporting polypeptide (OATP) family (32, 33) and the heterodimeric L-type amino acid transporters (LATs). Intracellular TH availability is regulated in a dynamically and tightly coordinated manner by specific deiodination processes. Deiodinases type 1and 2 (D1 and D2) activate THs whereas D3 carries out inactivation of T4 and T3 (34). Lastly, THs can either positively or negatively regulate gene transcription via the thyroid hormone receptor (THR) by binding to thyroid hormone response elements (TREs) located on the promotors of target genes or even via direct modulation of gene expression (35). THRs are encoded by the thyroid hormone receptor α (THRA) and thyroid hormone receptor β (THRB) genes each with specific transcriptional responses (36, 37).

Clinical assessment of thyroid function requires measurement of TSH. Elevated levels of TSH (and most often) simultaneously low TH indicate hypothyroidism, whereas suppressed TSH and high TH suggest a hyperthyroid condition. Subclinical hypo- and hyperthyroidism are characterized by TH values in the reference range and, respectively, high or low TSH (38). TSH is currently considered to be the most sensitive indicator of thyroid status, although assays and interpretations of thyroid function tests are not always straightforward, especially in the case of discordant results, notably with T4 or FT4 (39, 40).

Epidemiological studies report associations between TSH and/or TH as a function of exposure to numerous persistent as well as non-persistent pesticides. Similarly, experimental evidence suggests that pesticides may act as thyroid disruptors, affecting the HPT axis at several levels: central regulation, iodine uptake, production and distribution of THs, or binding of TH to membrane transporters or receptors (41–43). As a consequence, pesticide disruption of the HPT axis is increasingly scrutinized in Europe and in the United-States. In 2013, the European Food Safety Agency (EFSA) reported that 101 of the 287 pesticides they examined had the potential to interfere with thyroid function (44). As to the socio-economic costs of EDCs, in 2015 the neurobehavioral deficits (principally IQ loss) and neurodevelopmental diseases including attention deficit/hypoeractivity disorder (ADHD), induced by three substances with endocrine disrupting properties and acting principally on thyroid signaling, was estimated at 157 billion euros in the European Union (EU) (29). Of this 157 billion, 120 billion were attributed to organophosphate pesticides, such as chlorpyrifos.

**Figure 1:**
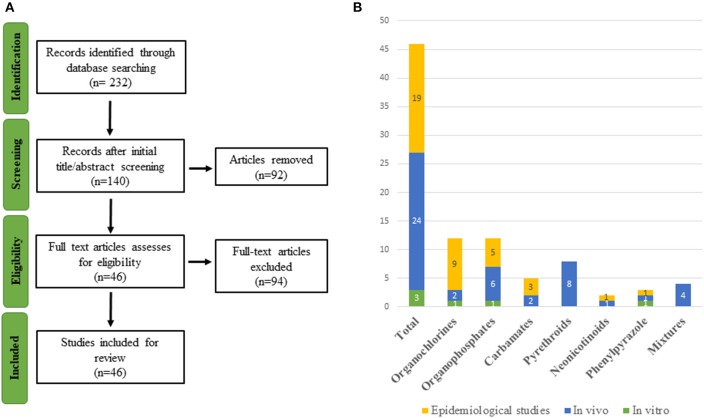
**(A)** Flowchart of study selection. **(B)** Graphical representation of the 46 included studies organized by compound and type of study.

Several reviews have shown that many xenobiotics from different chemical classes, including PPPs, are potential TH axis disrupting compounds acting at different levels of the HPT-axis (45, 46) but none has recently addressed different pesticide classes. Current knowledge regarding the full impact of pesticides on human thyroid function is still limited (47–49). To fill this gap, we reviewed the latest research on thyroid-related endpoints associated with old and newly formulated pesticides. The strategy is shown in Figure 1 and is detailed in the Methods section as well as in Supplementary Material.

## Methods

A literature search of scientific literature was carried out using PubMed (on the 21st of March 2019) for references on pesticides and thyroid endpoints. Studies that contained both a pesticide term and a TH outcome in the title and/or abstract were included. A total of 232 articles were retrieved.

Search terms combined an extensive list of thyroid endpoints and pesticide-related terms varying from broad (e.g., insecticides) to more specific (e.g., permethrin) terms. Chemical groups of pesticides were based on those widely used and we added certain substances from the EU's Pesticides Database^5^ Yale MeSh Analyser^6^ was used to add specific Mesh terms possibly omitted during preliminary searches. Searches compiled two parenthetical terms (TH outcome/pesticides) with an AND operator. These parenthetical terms contained sub-terms linked to each other via OR operators. The final search query is consultable in Table S1.

As the major aim of this review was to provide recent research conducted on pesticide exposure with thyroid-related adverse outcomes, the search in PubMed was limited to the last 5 years and involved two screening stages. A first selection was conducted on the title and abstract, and the second screening was based on full-text analysis. When uncertainty arose regarding the eligibility of a publication from its abstract, the full-text version of the article was retrieved to ensure that there was no inappropriate selection, e.g., “organochlorine” includes a diverse family of chemicals, some of which are not used as pesticides. The study selection was restricted to English-language articles. After analysis of title and abstract, 140 publications were retained for further analysis based on the full article. After excluding research that failed to meet the inclusion norms, a total of 46 publications was selected. The flowchart of the literature selection is presented in Figure 1. The references retrieved are organized by eligibility and classes of pesticides in an excel file accessible in Table S1.

In order to provide a more exhaustive overview, reference lists of included articles were also screened by title and abstract in order to include additional relevant articles without restrictions on their date of publication.

## Organochlorine Pesticides

OC pesticides (OCPs) are chlorinated hydrocarbon compounds, which were used extensively worldwide from the 1940s to the 1960s. They are considered to be the first generation of synthetic broad-spectrum pesticides, of which the most notable representatives are the insecticide DDT and the fungicide Hexachlorobenzene (HCB). OCPs represent a large class of chemicals which can be divided into dichlorodiphenylethanes (DDT, dicofol, and methoxychlor), hexachlorocyclohexanes (HCHs such as HCB, chlordane, lindane), cyclodienes (aldrin, dieldrin, endrin, endosulfan, heptachlor), and toxaphene, a complex mixture of highly chlorinated bornanes, as well as mirex and its derivative chlordecone (50–52). Their action stems from their capacity to alter ion exchange in nerve axons in the peripheral and central nervous system, resulting in decreased action potentials but the precise mechanisms of action are diverse and remain mostly unknown. Cyclodienes have the added effect of competitively binding to the GABA-A receptor (53).

DDT was widely applied to both US military and civilian populations during the Second World War to combat malaria, typhus and other insect-borne diseases (54). Given its efficiency and low-cost, DDT was excessively applied in the agricultural sector for crop and livestock protection, as well as in homes and gardens (54). In the 1950s, production reached 100,000 tons per year in the US (55) until Carson's Silent Spring (56) brought OCP's toxic effects on non-target wild-life species as well as humans to the fore of public scrutiny. Public concern led to the creation of the US EPA in 1970 and shortly after, the ban of many OCPs for agricultural use. In 2001, the international Stockholm Convention singled out 12 compounds as Persistent Organic Pollutants (POPs), 9 of which were OCPs [UNEP (57), “12 initial POPs”]. Despite the ban, OCs remain a widespread environmental pollutant due to their resistance to environmental degradation, bioaccumulation in lipid-rich tissues, and biomagnification in the food chain (58, 59).

### Epidemiological Evidence of Thyroid Disruption

Because DDT, HCB, and other OCPs have been associated with impaired neurodevelopment and neurocognition in infants and children (60–69), numerous epidemiological studies have investigated whether these adverse effects could be mediated, at least in part, by disruption of the HPT axis in either pregnant women or newborns (70–72, 72–81). An overview of the epidemiological organochlorine studies can be found in Table 1.

**Table 1:** Parameters of epidemiological data retrieved in the review – Organochlorines.

									**Thyroid hormone**		**Pesticide**										
**Chemical class**	**Study**	**Collection**	**Country**	**City**	**Population**	***N***	**Mean age (years)**	**Male-Female**	**Matrix**	**Time**	**Matrix**	**Time**	**Pesticide name**	**Mean concentration**	**TSH**	**TT3**	**FT3**	**TT4**	**FT4**	**Hypothyroidism**	**Other observations**
**EPIDEMIOLOGY—ORGANOCHLORINES**																					
Organochlorine	Takser et al. (78)	–	Canada	Southwest Quebec	Pregnant women and newborns	149	Maternal age: 27	0–100%	Serum and cord blood	1st, 2nd trimester, and delivery	Plasma and cord blood	1st, 2nd trimester, and delivery	Oxychlordane	1st trimester: 20 ng/L 2nd Trimester: 30 ng/L Delivery: 30 ng/L Cord blood: 10% detected	ns	ns	–	–	ns	–	TT3 levels during pregnancy were reduced in women exposed to five pollutants (PCB-138, PCB-153, PCB-180, p,p′-DDE, and HCB)
													*trans*-Nanochlor	1st trimester: 30 ng/L 2nd Trimester: 40 ng/L Delivery: 50 ng/L Cord blood: 14% detected	ns	ns	–	–	ns	–	
													*cis*-Nanochlor	1st trimester: 0% detected 2nd Trimester: 1% detected Delivery: 20% detected Cord blood: 0% detected	ns	↓ during pregnancy	–	–	ns	–	
													Mirex	1st trimester: 19% detected 2nd Trimester: 15% detected Delivery: 20% detected Cord: blood: 1% detected	ns	ns	–	–	ns	–	
													HCB	1st trimester: 40 ng/L 2nd Trimester: 60ng/L Delivery: 60ng/L Cord blood: 20 ng/L	ns	↓ during pregnancy	–	–	ns	–	
													β-HCH	1st trimester: 30 ng/L 2nd Trimester: 40 ng/L Delivery: 50 ng/L Cord blood: 1% detected	ns	ns	–	–	↓ during pregnancy	–	
													DDT	1st trimester: 10 ng/L 2nd Trimester: 30 ng/L Delivery: 40 ng/L Cord blood: 11% detected	ns	ns	–	–	ns	–	
													p,p′-DDE	1st trimester: 380 ng/L 2nd Trimester: 430 ng/L Delivery: 470 ng/LCord blood: 160 ng/L	ns	↓ during pregnancy	–	–	ns	–	
Organochlorine	Lopez-Espinosa et al. (82)	2003–2005	Spain	Valencia	Pregnant women	157	Maternal age: 30	0–100%	Serum	12 weeks of pregnancy	Serum	12 weeks of pregnancy	p,p′-DDE	1.3 ng/L	↑	ns	–	–	↓ during pregnancy	–	–
Organochlorine	Luo et al. (77)	November 213–June 2014	China	Hospitals in Henan	Pregnant women	115	Maternal age: 26.62	0–100%	Cord plasma	At birth	Cord plasma	At birth	a-HCH	0.24 ng/mL	ns	–	ns	–	ns	–	–
													β-HCH	0.62 ng/mL	ns	–	ns	–	ns	–	–
													g-HCH	0.31 ng/mL	ns	–	ns	–	ns	–	–
													d-HCH	0.63 ng/mL	ns	–	ns	–	ns	–	–
													Sum HCHs	0.0062 ng/mL	ns	–	ns	–	↓	–	–
													DDE	1.91 ng/mL	ns	–	ns	–	↓	–	–
													DDD	0.09 ng/mL	ns	–	ns	–	ns	–	–
													DDT	4.89 ng/mL	ns	–	ns	–	ns	–	–
													Sum DDTs	0.0201 ng/mL	↑	–	ns	–	ns	–	–
													Aldrin	8.58 ng/mL	↑	–	ns	–	ns	–	–
													Dieldrin	6.23 ng/mL	↑	–	ns	–	ns	–	–
													Endrin	0.49 ng/mL	ns	–	ns	–	ns	–	–
													Sum Drins	0.0412 ng/mL	↑	–	ns	–	ns	–	–
													Endosulfan I	2.62 ng/mL	ns	–	ns	–	ns	–	–
													Endosulfan sulfate	0.26 ng/mL	ns	–	ns	–	ns	–	–
													Sum endosulfan	0.0071 ng/mL	ns	–	ns	–	ns	–	–
													Methoxychlor	0.98 ng/mL	ns	–	ns	–	↓	–	–
													Heptachlor	1.45 ng/mL	ns	–	ns	–	ns	–	–
													Sum OCPs	0.0812 ng/mL	↑	–	ns	–	ns	–	–
Organochlorine	Asawasinsopon et al. (71)	2003–2004	Thailand	Mae Rim District of Chiang Mai Province	Pregnant women and newborns	39	Maternal age: 23.8	46.2–53.8%	Cord serum	At delivery	Cord serum	At delivery	p,p'-DDT	77.7 ng/g	ns	–	–	↓	ns	–	–
													p,p'-DDE	742 ng/g	ns	–	–	↓	ns	–	
													p,p'-DDD	89.1 ng/g	ns	–	–	ns	ns	–	
													o,p'-DDE	46.6. ng/g	ns	–	–	↓	ns	–	
													o,p'-DDT	17.1 ng/g	ns	–	–	ns	ns	–	
													Dieldrin	94.9 ng/g	ns	–	–	ns	ns	–	
													Heptachlor	37.1 ng/g	ns	–	–	ns	ns	–	
													Heptachlor epoxide	38.8 ng/g	ns	–	–	ns	ns	–	
POPs	Kim et al. (76)	February and December 2011	Korea	Seoul, Anyang, Ansan, Jeju	Pregnant women and newborns	148	Median maternal age: 33	0–100%	Umbilical cord serum + bloodspot newborn	Delivery and 2 days after birth	Cord blood and maternal serum	Delivery	Sum HCHs	Cord blood: 10.4 ng/g	ns	ns	ns	ns	ns	–	–
													Sum HCHs	Maternal serum: 9.4 ng/g	ns	ns	ns	ns	ns	–	–
													β-HCH	Cord blood: 7.5 ng/g	ns	ns	ns	ns	ns	–	–
													β-HCH	Maternal serum: 7.5 ng/g	ns	↓	↓	ns	ns	–	–
													Sum CHDs	Cord blood: 2.6ng/g	↑	ns	ns	ns	ns	–	–
													Sum CHDs	Maternal serum:3.9 ng/g	ns	ns	ns	ns	↓	–	–
													Sum DDTs	Cord blood: 65.2 ng/g	ns	ns	ns	ns	ns	–	–
													Sum DDTs	Maternal serum: 62.3 ng/g	↑ bloodspot	ns	ns	ns	ns	–	–
													p,p'-DDE	Cord blood: 63 ng/g	↑ bloodspot	ns	ns	ns	ns	–	–
													p,p'-DDE	Maternal serum:55.2 ng/g	↑ bloodspot	ns	ns	ns	ns	–	–
													HCB	Cord blood: 12.7.0 ng/g	ns	ns	ns	↓	ns	–	–
													HCB	Maternal serum: 5.5 ng/g	ns	ns	ns	ns	ns	–	–
Organochlorine	Maervoet et al. (79)	2002–2004	Belgium	Flanders	Pregnant women	198	Maternal age: 29.4	0–100%	Cord blood	Delivery	Cord blood	Delivery	p,p′-DDE	0.37 ng/mL	ns	–	ns	–	↓	–	–
													HCB	0.05 ng/mL	ns	–	↓	–	↓	–	
Organochlorine	Ribas-Fitó et al. (73)	1997–1999	Spain	Flix (catalonia)	Newborns	70	Not reported	Not reported	Plasma	3 days after birth	Cord serum	Delivery	p,p′-DDE	Not reported	↑	–	–	–	–	–	–
													HCB	Not reported	ns	–	–	–	–	–	–
													β-HCH	Not reported	↑	–	–	–	–	–	–
Organochlorine	Li et al. (83)	1997–2001	Denmark	Copenhagen	Placenta samples	58	Maternal age: 30.4	100% placenta's of boys	Placenta	Delivery	Placenta	Delivery	Sum 25 OCPs β-HCH methoxychlor	77.7 ng/g 9.18 ng/g 0.008 ng/g	– – –	ns ↓*p* < 0.081 ns	– – –	ns ↓*p* < 0.065 ns	– – –	– – –	Additionally measured rT3: methoxychlor were inversely associated with rT3
Organochlorine	Lopez- Espinosa et al. (72)	2004–2006	Spain	Valencia	Pregnant women and newborns	453	30	54.7–45.3%	Dry blood spot	3 days after birth	Cord serum	Delivery	p,p'-DDT	8.0 ng/g	ns	–	–	–	–	–	–
													p,p'-DDE	197 ng/g	ns	–	–	–	–	–	–
													HCB	75 ng/g	ns	–	–	–	–	–	–
													β-HCH	20 ng/g	↑*p* = 0.09	–	–	–	–	–	–
Organochlorine	Freire et al. (75)	2000–2002	Spain	Southern spain	Pregnant women and neonates	220	Maternal age: 31.8	100–0%	Cord blood	Delivery	Placenta	Delivery	o,p'-DDT	0.86 ng/g	ns	–	–	–	–	–	–
													p,p′-DDT	1.25 ng/g	ns	–	–	–	–	–	
													p,p′-DDE	2.01 ng/g	↑*p* = 0.09	–	–	–	–	–	
													o,p'-DDD	1.91 ng/g	ns	–	–	–	–	–	
													Sum DDTs	4.16 ng/g	ns	–	–	–	–	–	
													Endosulfan-I	0.73 ng/g	ns	–	–	–	–	–	
													Endosulfan-II	1.37 ng/g	ns	–	–	–	–	–	
													Endosulfan-diol	2.10 ng/g	ns	–	–	–	–	–	
													Endosulfan-ether	0.23 ng/g	ns	–	–	–	–	–	
													Endosulfan-sulfate	0.93 ng/g	↓	–	–	–	–	–	
													Endosulfan-lactone	1.14 ng/g	ns	–	–	–	–	–	
													Sum Endosulfans	4.02 ng/g	ns	–	–	–	–	–	
													Aldrin	0.82 ng/g	ns	–	–	–	–	–	
													Endrin	2.53 ng/g	↑	–	–	–	–	–	
													Dieldrin	1.05 ng/g	ns	–	–	–	–	–	
													Lindane	0.41 ng/g	ns	–	–	–	–	–	
													HCB	1.02 ng/g	↓*p* = 0.09	–	–	–	–	–	
													Methoxychlor	1.20 ng/g	ns	–	–	–	–	–	
													Mirex	1.15 ng/g	ns	–	–	–	–	–	
Organochlorine	Dufour et al. (81)	2013–2016	Belgium	Liege	Pregnant women and newborns	221	29.2	52.8–47.2%	Dry blood spot	3 days after birth	Cord serum	Delivery	HCB	0.0% detected	–	–	–	–	–	–	–
													β-HCH	0.5% detected	-	–	–	–	–	–	
													Trans-Nanochlor	0.0% detected	–	–	–	–	–	–	
													p,p'-DDE	24.1% detected	Boys: ↓	–	–	–	–	ns	
Organochlorine	Dallaire et al. (74)	1993–1996	Canada	Nunavik r (Quebec)	Pregnant women and neonates	410	Maternal age: 23	48.1–51.9%	Cord serum	Delivery	Cord plasma	Delivery	HCB	140 ng/L	ns	ns	–	–	↑	–	–
		1993–1997	Canada	Lower North Shore of the St. Lawrence River (Quebec)	Pregnant women and neonates	260	Maternal age: 25	48.5–51.5%	Cord serum	Delivery	Cold plasma	Delivery	HCB	150 ng/L	ns	ns	–	–	↑	–	
Organochlorine	Cordier et al. (80)	2004–2007	Guadeloupe	University Hospital Pointe-à-Pitre and the General Hospitals of Basse-Terre	Mother-child cohort	111	Maternal age: 30.7	0–100%	Child serum	At 3 months of age	Cord blood and breast milk samples	Cord blood: at delivery—breast milk: 3 months after delivery	Chlordecone	Median—cord blood: 0.14μg/L	Boys: ↑	–	ns	–	Boys: ns	–	–
															ns	–	ns	–	Girls: ns	–	–
														Breast milk	ns	–	Boys: ↓	–	Boys: ns	–	–
															ns	–	Girls: ↓	–	Girls: ↓	–	–
Organochlorine	Alvarez-Pedrerol et al. (70)	1997–1999	Spain	Island of Menorca	Children	259	Maternal age: 33	47.9–52.1%	Serum	At 4 years of age	Serum	At 4 years of age	p,p'-DDT	0.06 ng/mL	ns	↓	–	–	ns	–	–
													p,p'-DDE	0.88 ng/mL	ns	ns	–	–	ns	–	
													HCB	0.32 ng/mL	ns	ns	–	–	ns	–	
													β-HCH	0.22 ng/mL	ns	↓	–	–	ns	–	
Organochlorine	Meeker et al. (84)	January 2000 and May	North- America	Boston	Men	341	36	100–0%	Serum	Cross-sectional	Serum	Cross-sectional	p,p'-DDE	236 ng/g	↓	↑	–	–	↑	–	–
													HCB	15.6 ng/g	ns	↓	–	–	ns	–	–
		2003																			
Organochlorine	Bloom et al. (85)	2000–2002	North- America	Upper Hudson river communities	Women	48	63.2	0–100%	Serum	Cross-sectional	Serum	Cross-sectional	Sum DDT	3.59 μg/L	ns	↑	–	↑	ns	–	–
Organochlorine	Blanco-Munoz et al. (86)	July-October 2004 and December 2004–May 2005	Mexico	States of Mexico and Morelos	Floriculture workers (men)	136	32.7	100–0%	Serum	Longitudinal	DDE an DDT in serum and DAP metabolites in urine	Longitudinal study	DDE	6.14 and 4.71 ng/ml in rainy and dry seasons	ns	↑	–	↑	–	–	–
Organochlorine	Rathore et al. (87)	1997–1998	India	Jaipur	Women visiting the Thyroid Clinic	123	37	0–100%	Serum	Cross-sectional	Serum	Cross-sectional	Sum OC	18.83 ppm depleted T4 vs. 14.68 normal T4	ns	ns	–	ns	–	ns	–
													Total DDT (pp'DDE+pp' DDT+pp'DDD)	8.43 ppm depleted T3 vs. 6.91 normal T4	ns	ns	–	ns	–	ns	
													Total HCH (α,β,?)	3.82 ppm depleted T4 vs. 3.86 normal t4	ns	ns	–	ns	–	ns	
													Dieldrin	5.38 ppm in depleted T4 group vs. 2.5 normal T4	ns	ns	–	↓	–	↑	
													Hepatchlor	1.18 ppm in depleted T4 vs. 1.41 normal T4	ns	ns	–	ns	–	ns	
Organochlorine	Rylander et al. (88)	Not clearly indicated	Sweden	Swedish east coast, off the Baltic Sea	Fishermen	196	59	100–0%	Serum	Cross-sectional	Serum	Cross-sectional	p'p'-DDE	580 ng/g lipid	↑	–	–	–	ns	–	Also measured FSH, LH, estradiol, and testosterone Negative association: p,p'-DDE and estradiol level.
Organochlorine	Schell et al. (89)	1995–2000	North America	St. Lawrence River with territory in New York States, in Ontario and Quebec Canada	Mother–youth pairs	232	Youth: 17.6	–	Serum	Cross-sectional	Serum	Cross-sectional	HCB	Non-breast fed: 0.03 ppb breast-fed: 0.04	ns	ns	–	–	↓	–	Breast-fed adolescents had higher levels of p,p'-DDE
													p-p'-DDE	Non-breast fed: 0.31 ppb breast-fed: 0.41	ns	ns	–	–	ns	–	–
Organochlorine	Goldner et al. (48)	1993–1997 (Phase 1), 1999–2003 (Phase 2)	North America	Iowa, North Carolina	Female spouses of workers involved in Agricultural Health Study	16,529	47.2+HH109:L118	0–100%	Self- reported thyroid disease	Self- reported thyroid disease	Detailed self- reported use of pesticides.	Detailed self-reported use of pesticides.	Aldrin	–	–	–	–	–	–	↑	–
													Chlordane	–	–	–	–	–	–	↑	–
													DDT	–	–	–	–	–	–	↑	–
													Heptachlor	–	–	–	–	–	–	↑	–
													Lindane	–	–	–	–	–	–	↑	–
Organochlorine	Lerro et al. (92)	June 2010–September 2013	North- America	Iowa or North Carolina	Pesticide applicators	679	Not indicated	100–0%	Serum	Cross-sectional	Detailed self-reported use of pesticides.	Detailed self-reported use of pesticides.	Aldrin	–	↑	ns	–	↓	–	↑	–
													Chlordane	–	ns	ns	–	ns	–	ns	–
													DDT	–	ns	ns	–	ns	–	ns	–
													Heptachlor	–	ns	ns	–	ns	–	ns	–
Organochlorine	Piccoli et al. (90)	2012–2013	Brazil	Farroupilha, Serra gaucha, South Brazil	Agricultural workers	275	42	56.4–43.6%	Serum	Cross-sectional	Serum	Cross-sectional	HCH, HCB, heptachlor epoxide A, heptachlor epoxide B, heptachlor, transnonachlor, DDT, DDE, DDD, p,p'-DDD, endosulfan I, endosulfan II, aldrin, endrin, dieldrin, methoxychlor, mirex, pentachloroanisole	Many subject were below limit of detection, therefore no mean	Sum: ↓	↑	–	–	↑	–	–
Organochlorine	Shrestha et al. (91)	1991–1997	North- America	North Caroline and Iowa	Pesticide applicators	35,150	Median age 62	97.9–2.1%	Self- reported thyroid disease	Self- reported thyroid disease	Detailed self-reported us of pesticides	Detailed self- reported us of pesticides	Aldrin	Aldrin	nm	nm	nm	nm	nm	↑(attained age)	nm
													Heptachlor	Heptachlor	nm	nm	nm	nm	nm	↑(attained age)	nm
													Lindane	Lindane	nm	nm	nm	nm	nm	↑(attained age)	nm
													Chlordane	Chlordane	nm	nm	nm	nm	nm	↑ (all participants)	nm
Organochlorine	Goldner et al. (49)	1993–1997 (Phase 1), 1999–2003 (Phase 2)	North America	Iowa, North Carolina	Male private applicators (mainly farmers) in AHS	22,246	45.6	100–0%	Self- reported thyroid disease	Self- reported thyroid disease	Detailed self-reported use of pesticides.	Detailed self- reported use of pesticides.	Chlordane	–	–	–	–	–	–	↑	–
													DDT	–	–	–	–	–	–	↑	–
													Heptachlor	–	–	–	–	–	–	↑	–
													Lindane	–	–	–	–	–	–	↑	–
													Toxaphene	–	–	–	–	–	–	↑	–

A Canadian birth-cohort study (*n* = 101) studied several OCs in pregnant women and observed that p,p′-DDE, the main metabolite of DDT, HCB and a constituent of chlordane were negatively associated with total T3 (TT3) levels, and β-HCH with FT4 (78). At 12 weeks of pregnancy, higher concentrations of p,p′-DDE in maternal serum (*n* = 157) was associated with lower FT4 levels and higher TSH levels (82). In an exploratory cross-sectional study of 17 OCPs in neonates in China, Luo et al. (77) examined cord plasma concentrations (*n* = 115) of HCHs, p,p′-DDE and methoxychlor, and reported a negative association with FT4 levels. Other OCPs, such as aldrin and dieldrin, sum of DDTs and its metabolites, as well as the sum of OCPs were correlated with increases in TSH levels. In a small study of a farming population in northern Thailand (*n* = 39), cord serum levels of p,p′-DDT and p,p′-DDE were negatively associated with cord serum TT4 (71).

In a study on POPs in Korea (*n* = 104) by Kim et al. (76), β-HCH, chlordanes, DDT, and p,p′-DDE measured in mothers or in cord serum were associated with either decreased TH levels or increased TSH levels. Specifically, maternal p,p′-DDE was associated with decreased FT3, FT4, and TT4 in cord serum and was identified as a predominant determinate of bloodspot TSH with an interquartile range (IQR) increase of p,p′-DDE accounting for a 19% increase of TSH. Additional evidence of thyroid disruption was found in cord serum, with p′p-DDE associated with increased bloodspot TSH and decreased TT3. Maternal β-HCH was associated with decreased FT3 and TT3 in cord blood, while cord β-HCH was associated with increased bloodspot TSH. In cord serum, HCH was negatively associated with TT4. Maternal chlordanes were negatively associated with both cord fT4 and TT4 levels, and chlordanes in cord serum were positively associated with TSH.

A study in Belgium (*n* = 198) reported that, in cord plasma, HCB was associated with decreased FT3 and FT4, and p,p′DDE with decreased FT4, however no significant variations were detected for TSH (79). In a study on infants born in a HCB-polluted area in Spain (*n* = 70), Ribas-Fitó et al. (73) focused on TSH for determination of thyroid status. While no relationship was found for HCB, β-HCH, and p,p′-DDE were associated with higher TSH concentrations in plasma of neonates. Moreover, β-HCH tended to be negatively associated with TT3 (*P* < 0.065) and TT4 (*P* < 0.081) in placentas (*n* = 58) (83) and positively with TSH (*p* = 0.09) in cord serum (*n* = 453) (72).

Freire et al. (75), analyzed placenta samples (*n* = 220) from a male birth cohort in Spain and, out of 17 OCPs assessed, p,p′DDE and HCB in placenta presented a close-to-significant positive (*p* = 0.09) or negative association (*p* = 0.09) with cord blood TSH levels, respectively, and the cyclodiene endrin was associated with higher odds of increased TSH. In addition, the metabolite endosulfan-sulfate was related to lower TSH levels.

In contrast to previous studies, cord blood p′p-DDE levels (*n* = 221) were negatively associated with TSH levels when only male newborns were considered (81). Dallaire et al. (74) reported that prenatal exposure to HCB was positively associated with FT4 levels in newborns from two fish-eating populations in Quebec (*n* = 260 and *n* = 410). The authors considered, however, that nutrient confounders including iodine and selenium, may have mitigated the antagonistic effect of OCPs on TH. In 4 year old children (*n* = 259) which were followed in a Spanish birth cohort for examination of TH function, p,p′-DDT and β-HCH concentrations in serum were associated with decreased TT3 (70).

Chlordecone was banned in the USA in 1976 but was widely used until the 1993 in the French Caribbean (Martinique and Guadeloupe). In 2015, a longitudinal birth-cohort (*n* = 111) study in Guadeloupe investigated the effects of prenatal and postnatal exposure during breastfeeding. The authors reported that prenatal exposure was associated with increased levels of TSH in boys, while postnatal exposure determined at 3 months was associated with decreased FT3 levels in both genders, and FT4 levels in girls (80).

Taken together, the majority of mother/child cohort results show that OCPs may exert significant TH inhibitory effects. While several studies in new-borns and children are often considered unsatisfactory due to the lack of significant and concordant effects in both TH and TSH levels, given the high variability of each parameter, effects on either one can be considered to indicate a hypothyroid-like effect of many organochlorines.

When considering adults, results are more contradictory with some studies reporting effects more in line with hyperthyroidism in men (84), aging women (85) and occupational workers (86), while others found that OCPs were related to clinical or subclinical hypothyroidism in women (87), elderly men (88), adolescents (89) and pesticide applicators (48, 49, 90–92). Moreover, an analysis of data on several organochlorines acquired from the United-States cross-national survey NHANES^7^ did not find an effect of p,p′-DDE on TT4 in adults due to inconsistent results in both sampling cycles (1999–2000 and 2000–2001), and associations with TSH were not significant (93).

### *In vivo* Evidence of Thyroid Disruption From Animal Studies

*In vivo* studies have proposed several potential mechanisms underlying thyroid disruption by OCPs, majoritarily for DDT (63). Liu et al. (94) exposed male rats for 10 days to p,p′-DDE and reported a reduction in serum TT4 and FT4 along with decreased levels of transthyretin proteins responsible for T4 transport, upregulation of hepatic enzymes involved in T4 clearance as well as increased hippocampal thyroid hormone receptor mRNA. The effects observed encapsulate previously suggested modes of action of OCs. Many OCs may act by mimicking TH, binding to TRs along the HPT-axis, decreasing bioactivity of TH via increased clearance, and/or reducing binding to transport proteins due to their structural similarities to TH (95). Moreover, long-term exposure to low doses of DDT resulted in altered physiology and cytophysiological changes in the follicular epithelium of the thyroid gland to compensate for reduced secretion in thyrocytes (96, 97). Likewise, HCB disruption of the HPT-axis is characterized by decreased T4 in male rats, increases in hepatic T4-UDPGT activity along with increased T4 conversion to T3 in the thyroid and liver (98) and competitive inhibition of T4 binding to transporters by its major metabolite pentachlorophenol (PCP) (99, 100).

### *In vitro* Evidence of Thyroid Disruption

*In vitro*, a first line of evidence was the antagonistic action of DDT in a TSH-induced cAMP production assay (101), the principal second messenger required for thyroid gland activation. Rossi et al. (102, 103) investigated this mechanism of action and suggested that DDT could modify the lipid organization of the cell membrane and induce production of extracellular vesicles containing membrane bound TSH receptors (TSHR), thereby inducing failure of the TSH receptor to internalize and prolong TSHR-cAMP signaling. In addition, HCB was shown to reduce viability and inhibit cell cycle progression of FRTL-5 rat thyroid cells along with increased mRNA levels of transforming growth factor-beta (TGF-β1) known to inhibit cell growth in thyroid epithelial cells (104). In turn, modulation of TGF-β1 expression can have repercussions on thyroid function via regulation of thyroid specific genes such as those encoding NIS, thyroglobulin, thyroperoxidase, and the TSH receptor (105).

To conclude on OCPs, multiple mechanisms have been suggested to explain the reduction in circulating THs and/or the increase in TSH. These include displacement from distributor proteins, increased hepatic metabolism, and indirect effects on thyroid function.

## Organophosphate Pesticides

Organophosphates (OPs) pesticides (OPPs) are one of the two main classes of acetylcholinesterase inhibitors, the other being carbamates. During World War II, OPs were used for warfare as a human nerve gas agent, inducing seizures and, at high doses, respiratory arrest (106). Ultimately, OPs were adapted as insecticides because they act on insects via the same mechanism at relatively low doses. The adverse effects of OPs have been widely studied since the 1970s. Remarkably, even low-level prenatal exposure to various OPs, notably chlorpyrifos, can impede normal fetal brain development (107, 108). Despite the ban on in-house use in industrialized countries, chlorpyrifos, malathion, and diazinon continue to be extensively used for crop protection (109). After ingestion, most OP pesticides are metabolized, producing different dialkyl phosphate (DAP) metabolites which can be eliminated via urine within 24 h. As DAP metabolites can originate from different OPs, urinary DAP levels give an overview of general OP exposure rather than being a specific biomarker of a certain OP (110–112).

### Epidemiological Evidence of Thyroid Disruption

An epidemiological study conducted in California observed that prenatal, but not postnatal, levels of urinary DAP metabolites (*n* = 329) were associated with weaker intellectual development in 7-year-old children, highlighting the vulnerability of the developing fetus (113). In 2012, prenatal low-dose chlorpyrifos (CPF) exposure was associated with differences in brain morphology (107) but no TH related endpoints were measured. Authors of a small cross sectional study (*n* = 66) conducted on children in Indonesia, retrieved questionnaires, analyzed urine samples and measured classical thyroid related endpoints. Six different DAPs, originating from degradation of potentially 28 OPs, were measured in morning spot urine samples. The mean TSH level in children who tested DAP positive was significantly higher than those children with undetectable levels of DAP (114).

In adults, as for OCPs, less data is available. In an extensive prospective health study (*n* = 30,003), agricultural spouses were monitored and followed. The authors found that exposure to organophosphate insecticides was a risk factor for developing several hormonally-related cancers, including breast, thyroid, ovary and lymphoma (115). Further, a study conducted in China (*n* = 325) demonstrated that DAP levels in urine of pregnant women were positively associated with FT4 concentration and negatively with TSH levels, warranting further investigation on the consequences of OP exposure on thyroid function during pregnancy (116). An overview of TH-related epidemiological organophosphate studies can be found in Table 2.

**Table 2:** Parameters of epidemiological data retrieved in the review—organophosphates.

									**Thyroid hormone**		**Pesticide**										
**Chemical class**	**References**	**Collection**	**Country**	**City**	**Population**	***N***	**Mean age (years)**	**Male-Female**	**Matrix**	**Time**	**Matrix**	**Time**	**Pesticide name**	**Mean concentration**	**TSH**	**TT3**	**FT3**	**TT4**	**FT4**	**Hypothyroidism**	**Other observations**
**Epidemiology—Organophosphates**																					
Organophosphate	Suhartono et al. (114)	March–May 2015 and August–October 2015	Indonesia	Agricultural area, brebes destrict	Children from elementary school	66	9.2	52%-48%	Serum	Cross-sectional	Morning spot urine	Cross-sectional	6 DAP metabolites	Not indicated	↑	-	-	-	ns	↑ with positive urinary ogranophosphate pesticide metabolites	-
Organophosphate	Lerro et al. (115)	1993–1997	North-America	Iowa and North Carolina	Spouses of pesticide applicators	30,003	-	0%-100%	Detailed self-reported use of pesticides.	Detailed self-reported use of pesticides.	Detailed self-reported use of pesticides.	Detailed self-reported use of pesticides.	Detailed self-reported use of pesticides.	Detailed self-reported use of pesticides.	-	-	-	-	-	-	↑ risk with OP use for serval hormonally-related cancers including thyroid cancer
Organophosphate	Wang et al. (116)	April 2011-December 2013	China	Southern coastal area of Laizhou Wan (Bay) of Bohai Sea in Shandong Province	Pregnant women	325	28.4	0%-100%	Serum	cross-sectional	Urinary	cross-sectional	6 DAP metabolits: Sum DAP	-	↓	ns	ns	ns	↑	-	-
													DMP	9.81 μg/L	ns	ns	ns	ns	ns	-	-
													DMTP	0.79 μg/L	ns	ns	ns	ns	ns	-	-
													DEP	5.00 μg/L	ns	ns	ns	ns	ns	-	-
													DETP	0.78 μg/L	ns	ns	ns	ns	ns	-	-
50 different pesticides	Shrestha et al. (91)	1991–1997	North-America	North Caroline and Iowa	Pesticide applicators	35,150	Median age 62	97.9%-2.1%	Detailed self-reported use of pesticides.	Detailed self-reported use of pesticides.	Detailed self-reported use of pesticides.	Detailed self-reported use of pesticides.	Diazinon	-	-	-	-	-	-	↑(attained age)	-
													Dichlorvos	-	-	-	-	-	-	↑(attained age)	-
													Malathion	-	-	-	-	-	-	↑(attained age)	-
													Glyphosate	-	-	-	-	-	-	↑(attained age)	-
50 different pesticides	Goldner et al. (91)	1993–1997	North-America	North Caroline and Iowa	Pesticide applicators	22,246	45.6	100%-0%	Self-reported thyroid disease	Self-reported thyroid disease	Detailed self-reported use of pesticides.	Detailed self-reported use of pesticides.	Diazinon	-	-	-	-	-	-	↑	-
													Malathion	-	-	-	-	-	-	↑	-
													Glyphosate	-	-	-	-	-	-	ns	-
Total of 33 pesticides (16 herbicides, 13 insecticides, two fungicides, two fumigants)	Lerro et al. (92)	June 2010-September 2013	North-America	Iowa or North Carolina	Pesticide applicators	679	Not indicated	100%-0%	Serum	Cross-sectional	Detailed self-reported use of pesticides.	Detailed self-reported use of pesticides.	Chlorpyrifos	-	ns	ns	-	ns	-	ns	-
													Diazinon	-	ns	ns	-	ns	-	ns	-
													Fonofos	-	ns	ns	-	ns	-	ns	-
													Malathion	-	ns	ns	-	ns	-	ns	-
													glyphosate	-	ns	ns	-	ns	-	ns	-

### *In vivo* Evidence of Thyroid Disruption From Animal Studies

*In vivo*, a mouse study reported that a 3-day administration of CPF at doses under the concentration required for inhibition of brain acetylcholesterinase, could induce histological and histo-morphometrical thyroid effects, resulting in hypothyroidism in dams. In the F1, exposed during pre- (3 days) and post-natal (additional 3 days) periods, long-term reductions in serum T4 levels were measured in males 150 days after birth (117). Despite the concentration levels of CPF in this study being much higher than the levels estimated in children (118), these results may still be of significance due to potential additive effects that are likely to occur from continuous exposure to a variety of organophosphorus compounds (see section Mixtures). In addition, Mie et al. (119) reported that the manufacturers' report that was submitted for authorization of chlorpyrifos was misleading as significant effects of exposure on brain development were omitted from conclusions. Specifically, cerebellum height and brain weight were reduced at intermediate doses. As TH is essential for Purkinje cell differentiation in the cerebellum, a thyroid disrupting effect could be hypothesized.

CPF interferes with maturation of surgeonfish (*Acanthurus triostegus*) through a thyroid hormone-dependent process. By inhibiting TH levels, chlorpyrifos disrupted metamorphosis and reduced the ability of juveniles to graze algae, an important factor in coral reef maintenance (120). Another fish study conducted a 21-day exposure of OP monocrotophos (MCP) to goldfish (*Carassisu auratus)*. Exposure concentrations were 0.01, 0.10, and 1.00 mg/L. Expression profiles of the HPT axis-responsive genes were altered in the liver, brain, and kidneys, and plasma levels of T3 were decreased (121). This study was repeated in 2018 (122) using lower concentrations of MCP (0, 4, 40, and 400 μg/L) and a different exposure protocol (2-, 4-, 8-, and 12-days of exposure) and confirmed the TH disrupting properties of MCP. Also, an economically important teleost species, the Caspian roach (*Rutilus rutilus)* appears to be vulnerable to organophosphate exposure. Research has shown that when young Caspian roach were exposed to environmentally relevant concentrations of diazinon for 9 h, levels of TSH, T4 and T3, were significantly reduced. Furthermore, cortisol and glucose levels were significantly increased. These alterations of physiology might impact survival rates leading to restocking failures through dwindling numbers of juveniles released into river estuaries (123). Ortiz-Delgado et al. (124) investigated malathion exposure effects in the Senegalese sole, *Solea senegalensis*, a flatfish whose morphology, obtained gradually through metamorphosis, is TH-dependent. By exposing fish to malathion (another OPP), the authors demonstrated the sensitivity of this process to this pesticide that is still widely used in developing countries.

### *In vitro* Evidence of Thyroid Disruption

Qiu et al. (125) used FRTL-5 cells to investigate effects of malathion on TH biosynthesis and showed decreased TSH receptor expression. Toxicogenomics from *in vitro* experiments aim to identify molecular patterns able to predict *in vivo* adverse outcomes. This strategy responds to the urgent need for a rapid mechanism-based strategy in risk assessment. In line with this concept, transcriptome analysis was conducted on immortalized rat thyrocytes that were exposed to either CPF or ethylenethiourea (ETU) in order to define *in vivo* gene signatures and mechanisms of toxicity. They showed non-monotonic dose response curves for both compounds along with common and distinct effects on thyroid toxicity, including altered growth of thyrocytes after chemical exposure to either ETU or CPF. Gene expression based on *in vivo* experiments fell short of fully recapitulating *in vitro* predictions because of compensatory and feedback loop mechanisms that are active *in vivo*. Despite this limitation, *in vitro* toxicogenomics managed to predict modes of action with longer exposure times. Notably, interference with thyrotrope growth was the main mechanism identified (126).

To conclude on the diverse mechanisms underlying actions of different OPPs on thyroid equilibrium. Both malathion and CPF could affect thyrotrope production, leading to TH lowering effects. However for CPF, even though the brain effects. On the other hand, for chlorpyrifos, even though the brain effects have been clearly demonstrated in rats and the TH lowering effects are well-identified in fish, the actual mode of action remains to be clarified *in vivo*.

### Glyphosate

Glyphosate is an OP compound used worldwide as a broad-spectrum herbicide. It binds and competitively inhibits the activity of enolpyruvylshikimate-3-phosphate synthase (EPSPS), an enzyme involved in the shikimic acid pathway (127) only found in plants and micro-organisms (128). Glyphosate usage has increased tremendously since Monsanto introduced glyphosate-tolerant crop varieties in 1996 (namely, sugar beet, canola, cotton, maize, alfalfa) to be used in conjunction with Roundup, the glyphosate-based formulation (129). The median half-life of glyphosate in the field is reportedly 47 days and the primary breakdown products are aminomethylphosphonic acid (AMPA) and glyoxylate (130).

#### Epidemiological Evidence of Thyroid Disruption (Three Studies)

A prospective cohort study of licensed pesticide applicators in North Carolina and Iowa with 35,150 male and female participants demonstrated that self-reported use of glyphosate was associated with increased risk of hypothyroidism (91). However, these findings are not consistent with results obtained from the same cohort study conducted by Goldner et al. (49) and Lerro et al. (92).

#### *In vivo* Evidence of Thyroid Disruption

Surprisingly, few studies retrieved were found to be relevant to glyphosate exposure and thyroid endpoints. In a first i*n vivo* study, female pregnant Wistar rats were exposed to either 5 or 50 mg/kg/day Roundup®Transorb (Monsanto) from gestation day 18 to post-natal day (PND) 5. Blood and tissues samples from heart, liver, pituitary and hypothalamus were collected for hormonal, metabolomics or gene expression analysis at PND 90. In every tissue collected, TH-related genes were observed to be differentially expressed in exposure groups compared to the control group. Levels of TSH were decreased in exposure groups, however levels of both T3 and T4 were unaffected. This curious lack of TH hormone effects, despite TSH and target gene changes, may reflect changes in TSH set-point resulting from differential gene programming in rats during the fetal period (131).

Due to the removal of patent protection for glyphosate in 2000, many new glyphosate-based herbicides arrived on the pesticide market. Each of these formulations have a slightly altered surfactant mixture and chemistry, making testing procedures even more complicated. The second *in vivo* study, exposed four different North American amphibian species *(Rana clamitans, Rana pipiens, R. sylvatica*, and *Bufo americanus)* to glyphosate and the surfactant polyethoxylated tallowamine (POEA), and six different glyphosate-based formulations. Disruption of the HPT-axis was investigated by measuring time to metamorphosis and expression of *thrb*. Glyphosate alone and formulations lacking POEA were the least toxic, however *R. pipiens* tadpoles showed delayed metamorphosis and decreased snout-vent length at the peak of metamorphosis after exposure to either POEA or glyphosate formulations containing POEA. These effects may be linked to the increased *thrb* mRNA levels observed in the same exposure conditions. This study underlines the need for surfactant composition to be taken into consideration in the evaluation of risk assessment of glyphosate-based herbicides (132). In another frog study (*Lithobates sylvaticus*), glyphosate-formulated exposure alters brain gene expression for *thrb* and *dio3* enzyme, with different alterations depending on the stage (133).

Clearly, how and at which levels glyphosate has the potential to interfere with TH equilibrium in different species has not yet been fully examined. However, one area that remains to be investigated is microbiome metabolism. As human, vertebrate and invertebrate microbiomes express the EPSPS enzyme, it is plausible that microbiome status is modified by short or long-term glyphosate exposure.

## Carbamates

Carbamates are widely used in agriculture, principally as insecticides, but also as herbicides and fungicides. Although they differ chemically from organophosphates, they act similarly by inhibiting the acetylcholineresterase enzyme (AChE) at the level of neuronal synapses. AChE is responsible for the rapid hydrolytic degradation of the neurotransmitter ACh into inactive products at neuromuscular junctions. In general, the AChE inhibition by carbamates is reversible in contrast with OPs (134). We focused here on the subgroup of dithiocarbamates in which, both oxygen atoms are replaced by sulfur. Dithiocarbamates can be sub-divided into two major groups: ethylenebisdithiocarbamates (EBDC) which includes maneb, zineb, and mancozeb, and dimethyldithiocarbamates (DMDC) consisting of ferbam, ziram and thiram. ETU is one of the major metabolites of EBDCs in mammals (135) whereas carbon disulfide is a metabolite found after *in vivo* DMDC treatment (136). As the thioureas can inhibit thyroid peroxidase, a thyroid gland enzyme essential for TH production, pesticides that generate ETU metabolites are of particular concern (55).

### Epidemiological Evidence of Thyroid Disruption

Chronic exposure to EBDCs mainly concerns agricultural and industrial workers but also the general population which may be continuously exposed to residues present in food (137). An overview of the epidemiological carbamate studies can be found in Table 3. In Costa Rica, mancozeb is applied weekly on banana plantations by light aircraft. Urinary ETU concentrations of pregnant women living in the vicinity of plantations (*n* = 451) were more than five times higher than concentrations reported in general populations (138). In 2017, a large epidemiological study was conducted in Taiwan to investigate the association between hypothyroidism and anticholinesterase pesticide poisoning (organophosphate and carbamate). A total of 10,372 subjects poisoned by anticholinesterase pesticides were compared to 31,116 reference subjects between 2003 and 2012. Analysis demonstrated that exposed subjects had significantly increased risk for hypothyroidism (139). A smaller study was conducted on 177 occupationally exposed male workers in Italy and confirmed the thyroid disrupting effect of mancozeb (140).

**Table 3:** Parameters of epidemiological data retrieved in the review—carbamates.

									**Thyroid hormone**		**Pesticide**										
**Chemical class**	**References**	**Collection**	**Country**	**City**	**Population**	***N***	**Mean age (years)**	**Male-Female**	**Matrix**	**Time**	**Matrix**	**Time**	**Pesticide name**	**Mean concentration**	**TSH**	**TT3**	**FT3**	**TT4**	**FT4**	**Hypothyroidism**	**Other observations**
**Epidemiology—Carbamates**																					
Carbamates	Van Wendel de Joode et al. (138)	March 2010 and June 2011	Costa rica	Matina County, Limon	Pregnant women	415	24	0–100%	nm	nm	Urine	ETU	Main metabolite of mancozeb: ETU	4.2 μg/L	nm	nm	nm	nm	nm	nm	ETU concentration > than 5 times ↑ than those reporterd for other general populations
Carbamates and organophosphate	Huang et al. (139)	2003–2012	Taiwan	Nationwide	ACPP subjects and non-ACPP* population-based	ACPP = 10.372 non-ACPP = 31.116	54.27	72.13–27.87%	nm	nm	Comparison anitocholestinerase pesticide poisoning with non-pesticide poisoning subjects		Anticholinesterase pesticide poisoning		nm					↑ risk	nm
Carbamates	Medda et al. (140)	July–August	Italy	Chianti area and Bolzano province	occupationally exposed grapevine workers (male)	177	44.6	100–0%	Serum, urinary	Cross-sectional	Plasma	Cross-sectional	ETU	12.2 μg/L	ns	nm	↑	↓	↑	nm	Additionally urinary iodine and thyroglobulin was measured in serum but no significant results were obtained

### *In vivo* Evidence of Thyroid Disruption From Animal Studies

A rat study conducted in 1985 demonstrated that both EBDCs maneb and zineb could affect endogenous TRH at the pituitary or hypothalamic level and therefore inhibit TSH secretion (141). At the level of the thyroid gland, Mancozeb was shown to reduce TPO activity, consequently altering both weight and histopathology of the thyroid gland, with acute high dose exposure resulting in a hypothyroid status in rats (142, 143).

A zebrafish study exposed embryos to 0.001–10 μM thiram, at various developmental stages for a short duration of time (1 h). Thiram exposure increased *dio3* mRNA expression a 12 hpf and decreased *tpo* mRNA expression at 48 hpf. In addition, delayed hatching, increased mortality, and skeletal defects were observed. The extent to which the disruption of the HPT axis contributed to these specific adverse outcomes needs further investigation (144).

### *In vitro* Evidence of Thyroid Disruption

The effects of ziram, thiram, zineb, and ETU have been investigated *in vitro*, on Chinese hamster ovary cells transfected with the human *TPO* gene. Zineb (50 μM) inhibited the iodinating activity of TPO at a 10-fold higher concentration than its metabolite, ETU, indicating that ETU is the ultimate toxicant *in vivo*. No effect was observed with DMDCs, thiram and ziram (145).

To conclude on carbamates, a major concern is the effects of many metabolites on TPO activity. A major gap to be filled is that there is little epidemiological data available on exposure levels in the environment and general population.

## Pyrethroids

Pyrethrins are found in Chrysanthemum flowers and serve as a natural system of protection against insects (146). One major limitation of organic pyrethrins is their fast photo-degradation leading to limited usage in agriculture. Hence, researchers established a more stable synthetic compound, labeled pyrethroids (PYRs), based on the chemistry of natural pyrethrins. The chemical structures of PYRs are comparable across the group and retain the essential acid/alcohol configuration of pyrethrins. Both groups block normal nerve impulses by preventing closure of voltage-gated sodium channels in axonal membranes, thereby paralyzing and eventually killing the organism (147).

Currently, PYRs represent a major class of insecticides worldwide, with more than 14% of the total pesticide market in 2005 (148). The most common PYRs include allethrin, bifenthrin, cyfluthrin, λ cyhalothrin, cypermethrin, deltamethrin, permethrin, d-phenothrin, fenvalerate, resmethrin, and tetramethrin (149). Two frequent metabolites, 3-(2,2-dichlorovinyl)-2,2-dimethylcyclopropne carboxylic acid (DCCA) and 3-phenoxybenzoic acid (3-PBA), are non-specific metabolites of several different PYR insecticides (150).

The major route of exposure for humans is through ingestion of food items treated with PYRs (151). Even though PYRs have relative short half-lives ranging from 3- to 96-days in soil (152), they are found in environmental samples (153, 154), human samples (155) and food (156), probably as a result of excessive and repeated use. Given PYRs structural resemblance to T3 and T4, PYRs insecticides are suspected to act as TH disruptors (150). An overview of the epidemiological PYR studies can be found in Table 4.

**Table 4:** Parameters of epidemiological data retrieved in the review—pyrethroids.

									**Thyroid hormone**		**Pesticide**										
**Chemical class**	**Reference**	**Collection**	**Country**	**City**	**Population**	***N***	**Mean age (years)**	**Male-Female**	**Matrix**	**Time**	**Matrix**	**Time**	**Pesticide name**	**Mean concentration**	**TSH**	**TT3**	**FT3**	**TT4**	**FT4**	**Hypothyroidism**	**Other observations**
**Epidemiology—Pyrethroids**																					
Pyrethroids	Zhang et al. (157)	2009–2011	Japan	University Hospital Tokyo	Pregnant women	231	34.1	0–100%	Serum	10-12th week of pregnancy	Urine	10-12th week of pregnancy	3-PBA	0.363μg/g	ns	-	-	-	ns	-	Also measured thyroid binding globuline but no significant results were obtained

### Epidemiological Evidence of Thyroid Disruption

As determined by urinary levels of metabolites to estimate internal exposure, human exposure to PYRs is widespread (155). Pregnant women are of particular concern given the vulnerable window of prenatal exposure to pesticides that could lead to impairment of normal development of offspring. The most commonly detected PYR metabolite is 3-PBA, a breakdown product of several PYR, that has been repeatedly measured in urine of pregnant women. However, no association was found between maternal TH serum levels and PYR exposure (157). Concealed behind this outcome remain important facts that should be taken into consideration: (i) both PYRs and their metabolites have the capacity to induce antagonistic effects on TRs (ii) they are able to transfer to the placenta (150, 158, 159). It might be prudent to carry out more regular measurements of TH levels in women exposed to PYRs and eventually examine offspring for TH related effects.

### *In vivo* Evidence of Thyroid Disruption From Animal Studies

In adult rats, two separate *in vivo* studies observed alterations of TH levels in serum using different PYRs and distinct exposure protocols (160, 161), suggesting that PYRs are potential thyroid disruptors. In another rodent study, pregnant mice were orally administered a PYR (fenvalerate) throughout pregnancy on a daily basis. Quantitative analysis of mRNA in the placenta of exposed mice showed a reduction of TRα1 and TRβ1 transcripts. Additionally, fetal intrauterine growth retardation (IUGR) was observed in offspring that were exposed during embryonic development. Following the observation of altered maternal THs together with IUGR, one may hypothesize that disruption of placental TR mRNA could explain poor fetal intrauterine growth (162). Furthermore, a study using lizards (*Eremias argus)* as a model organism showed that a 21-day exposure to λ -cyhalothrin alters the expression of TH-related genes in the liver (163). A zebrafish study demonstrated that permethrin, one of the most frequent pyrethroids, significantly increased expression of major thyroid signaling genes (thyroid hormone receptors, deiodinases, thyroid-stimulating hormone) as well as transthyretin (TTR) protein in zebrafish larvae, after a single 3-day embryonic exposure. Additionally, the same research group showed that permethrin has the potential to alter TTR activity by docking to TTR's active pocket (164). This research group also identified bifenthrin and λ-cyhalothrin as disruptors of the HPT axis in zebrafish embryos. The majority of the genes examined related to the HPT axis (*tpo, dio1, dio2, thra, thrb, ttr)* were found to be upregulated (165). Another zebrafish study tested two PYRs (permethrin and β-cypermethrin) and three metabolites: 3-phenoxybenzoic alcohol (PBCOH), 3-phenoxybenzaldehyde (PBCHO), and 3-phenoxybenzoic acid (PBCOOH). The study demonstrated that both PYRs and their metabolites exert effects on TH signaling, locomotor behavior and development of embryonic zebrafish. The observed thyroid disruption may play a role in the aberrant larval development (166).

An additional factor that should be taken into consideration is that fish are vulnerable to changes in temperature attributable to global climate change and that the increase in temperature might exacerbate the effect of chemical exposure. One study conducted by Giroux et al. (167) investigated both the effects of higher temperature and pyrethroid (bifenthrin) exposure on TH signaling. To better understand the possible interaction between pesticide exposure and temperature on salmon development, researchers reared the fish at different temperature (11, 16.4, and 19°C) and exposed them at different concentrations (0, 0.15, and 1.5 μg/L) of bifenthrin for 96 h. Final results revealed decreased survival with increasing temperatures. Following pesticide exposure, TH levels were either significantly increased in the case of juvenile exposure in warm water and tended to decrease during fry stages at low temperatures. These adverse sub-lethal effects could have long-term consequences for populations, such as those featured in this study in California which are affected by both a seasonal rainstorm runoff containing pyrethroid pesticides and increasingly warmer waters.

### *In vitro* Evidence of Thyroid Disruption

An *in vitro* research reported that an array of PYR insecticides (cycloprothrin, cyfluthrin, cyhalothrin, cypermethrin, deltamethrin, etofenprox, fenvalerate, permethrin, and tetramethrin) and their prevalent metabolite, 3-PBA, have the potential to disrupt TH signaling. By making use of a receptor-mediated luciferase reporter gene assay, Diu et al. (150) demonstrated that the aforementioned PYRs, of similar chemical structure, exert antagonistic action on the TH receptor and therefore impede the TH axis. The potential for PYRs and their metabolite to interact with androgen or estrogen receptor was also investigated. Interestingly, results suggested that, in the case of estrogen signaling, the metabolites rather than their parent compound should be given greater concern as they were up to 1,000-times more potent in their interaction with the receptor (150).

To conclude on pyrethrins, their structural resemblance to THs, with the fact that *in vitro* and *in vivo* animal studies demonstrate clear interference with TH homeostasis and action argues for more caution in their use and more intense scrutiny of their long-term effects.

## Phenylpyrazole

Fipronil, the most representative synthetic pesticide of the phenylpyrazole family, is a broad-spectrum insecticide and acaricide. This “second generation” pesticide is widely used in crop protection in urban areas for insect control and in veterinary practice for its efficiency against domestic animal ectoparasites (168). As fipronil usage has increased rapidly since its introduction in 1993—accounting for approximately 10% of the global pesticide market (169, 170), it has also become a widespread environmental contaminant detected in both soil and water (171, 172), indoor and outdoor dust (173) as well as in various food matrices as highlighted by the recent egg contamination scandal [(174, 175)^8^] Fipronil was partially restricted in 2013 and prohibited for outdoor use in most EU nations during flowering periods after its suspected involvement in the historical mass mortalities of honey bees (176). However, this decision was recently overturned by the European court due to insufficient impact assessment (177). Fipronil is still used as a biocide for ants and cockroaches as pests in the EU, but there has not been an application for its re-registration for use as a systemic pesticide in agriculture (178). Initially introduced after increasing resistance to organophosphate, pyrethroid and carbamate pesticides (170), its neurotoxicity arises from non-competitive blocking of gamma-aminobutyric acid (GABA)-gated chloride channels resulting in excessive neuronal stimulation and death. Its specificity for target organisms derives from its higher affinity for invertebrate GABA-A and GABA-C receptors than those of mammals (169, 179, 180).

Adverse effects were found in numerous non-target organisms including beneficial insects such as bees and termites, and vertebrates including fish, reptiles, birds, and mammals (168). In addition to hepatotoxic, nephrotoxic, anti-reproductive, and cytotoxic effects (180). Alarmingly, the primary metabolite fipronil-sulfone and the photodegradate fipronil-desulfinyl are both biologically active and are considerably more toxic, persistent, bioaccumulative, and less selective than the parent compound (181–187). Since fipronil is rapidly metabolized into the sulfone metabolite which has a longer half-life (208 h instead of 8.5 h in rats) and a six-fold greater binding capacity to vertebrate GABA receptors than fipronil (181), fipronil-sulfone has been suggested as the main mediator for fipronil-induced toxicity (180, 187, 188).

### Epidemiological Evidence of Thyroid Disruption

In human studies, serum fipronil-sulfone, the main metabolite of fipronil, was inversely correlated with serum TSH levels in factory workers manufacturing veterinary drugs containing fipronil, suggesting a central inhibitory effect on TSH secretion (189). While exposure of the general population is lower than occupational exposure, fipronil exposure can nonetheless affect sensitive populations such as newborn infants. In a pregnancy-birth cohort study, maternal fipronil-sulfone was reported to be placentally transferred to newborn infants along with exposure levels inversely correlated with FT3 levels in both cord blood and newborns (169). Furthermore, fipronil-sulfone concentrations were negatively associated with 5-min Apgar scores (a method used for assessing new born status immediately after birth and a biomarker of developmental vulnerability) (190). An overview of the epidemiological fipronil studies can be found in Table 5.

**Table 5:** Parameters of epidemiological data retrieved in the review—phenylpyrazole.

									**Thyroid hormone**		**Pesticide**										
**Chemical class**	**References**	**Collection**	**Country**	**City**	**Population**	***N***	**Mean age (years)**	**Male- Female**	**Matrix**	**Time**	**Matrix**	**Time**	**Pesticide name**	**Mean concentration**	**TSH**	**TT3**	**FT3**	**TT4**	**FT4**	**Hypothyroidism**	**Other observations**
**Epidemiology—Phenylpyrazole**																					
Phenylpyrazole	Herin et al. (189)	2008	France	factory manufacturing fipronil-containing veterinary drugs	Workers of factory	159	34.1	51% - 49%	Serum	Cross-sectional	Serum	Cross-sectional	Fipronil	0.47 mg/L	ns	-	-	ns	ns	-	-
													Fipronil sulfone	7.79 mg/L	↓	-	-	ns	ns	-	-
Phenylpyrazole	Kim et al. (169)	March 2013-July 2015	South-Korea	Inje University Ilsan Paik Hosptial	Pregnant women and matching biological fathers	169 participants, 59 mother-neonate pairs and 51 matching biological	32.08 age at delivery	100%-100%	Cord blood	At birth	Serum mother—serum father—cord blood	Delivery	Fipronil	Below levels of detection	ns	ns	ns	ns	ns	-	-
													Fipronil sulfone	Geometric mean fipronil sulfone—maternal serum: 0.744 ng/mL—paternal serum: 1.163 ng/mL—cord blood serum: 0.525 ng/mL	ns	Infantile fipronil sulfone levels were inversely associated with cord blood T3	Infantile fipronil sulfone levels were inversely associated with cord blood FT3	ns	ns	-	-

### *In vivo* Evidence of Thyroid Disruption From Animal Studies

Exposure to fipronil caused tumors in rats via hypertrophy of thyroid follicles (191) and altered the integrity of follicular cells, thyroid tissue and even the chemical composition of the colloid in mice (186, 192). Moreover, fipronil exposure decreased circulating TH levels in rats, at least partially mediated by increased clearance following elevated activity and expression of phase II hepatic enzymes (186, 187, 193, 194).

### *In vitro* Evidence of Thyroid Disruption

Using *in vitro* reporter gene assays, fipronil-sulfone showed antagonistic activity via TRβ in a dose-dependent manner, suggestive of another potential mechanism of action for the thyroid disrupting effect of fipronil (195).

## Neonicotinoids

Neonicotinoids are a relatively new class of broad-spectrum insecticides discovered in the 1980s and widely used today in agriculture for their systemic properties (170), but also in commercial, residential and veterinary settings (196). The global insecticide market, previously dominated by carbamates, organophosphates and pyrethroids was reshaped in 2008 when neonicotinoids represented over a quarter of the market. In 2008, the neonicotinoid imidacloprid (IMI) was the world's largest selling insecticide, second only to the herbicide glyphosate (170, 197). Currently, the main neonicotinoids on the market are imidacloprid, thiamethoxam, thiacloprid, clothianidin, acetamiprid, nitenpyram, and dinotefuran, however the first three were recently banned for outdoor use in Europe during flowering, following demonstrated risks to bees and other pollinators. Structurally similar to the natural insecticide nicotine—historically used for centuries as an early insecticide, neonicotinoids have enhanced selectivity and potency for the insect nicotinic acetylcholine receptor (nAChR), compared to vertebrate nAChR subtypes, and relatively poor penetration of the mammalian blood–brain barrier, translating into apparent reduced health risks for mammals, birds and fish (196, 198). The insecticidal activity of neonicotinoids originates from binding to nAchRs, triggering a large influx of cations in the postsynaptic membrane of nerve cells in the central nervous system, causing excessive excitatory neurotransmission which results in paralysis and death (198).

### Epidemiological Evidence of Thyroid Disruption

Although there is no epidemiology of TH levels in humans exposed to neonicotinoids, there is one report of reduced IQ as a function of prenatal exposure to neonicotinoids and pyrethyroids (199).

### *In vivo* Evidence of Thyroid Disruption

Pandey and Mohanty (142) examined potential thyroid disrupting effects in wild male finches exposed to IMI under laboratory conditions. They reported altered thyroid histopathology including increases in thyroid weight and volume, hypertrophy and hyperplasia of epithelial and stromal cells, decreases in plasma T4, T3 and TSH levels. It should be noted that a commercial formulation of IMI was utilized which contains multiple potentially bioactive ingredients (200).

### *In vitro* Evidence of Thyroid Disruption

A TH-dependent proliferation assay (T-screen) with GH3 cells was employed to investigate the thyroid disrupting potential of common neonicotinoids, but none of the 7 neonicotinoids tested had any effect on TH-dependent proliferation (200) in the absence of T3. However, Xiang et al. (201) showed that IMI exerted agonist effects in a GH3 luciferase reporter gene assay mediated via TR which was further characterized using human TRβ in a Surface Plasmon Resonance biosensor technique to measure binding kinetics. Moreover, *in silico* molecular docking (MD) analysis predicted that IMI may compete with T3 for binding with TR by forming hydrogen bonds with the imidazole group (202).

Overall, despite their widespread use, relatively few studies have investigated the potential thyroid disrupting effects of neonicotinoids, but many have examined the effects of neonicotinoids in mixtures containing various classes of pesticides, which is more in line with the reality of environmental exposure.

## Mixtures

Humans are simultaneously exposed to myriads of chemicals that may act in concert to induce mixture effects. Currently, risk assessment is focused on examining chemicals individually, without taking into account potential toxic effects of low-dose combinations of EDCs. Frequently, in agricultural practice, multiple pesticides are preferentially used to reduce resistance to an individual pesticide and enhance efficacy (203). In the case of combined exposure with one or more families of different pesticides, several modes-of-action could be involved. Furthermore, each chemical may interfere with another, resulting in complex dose-response interactions (204). It is important to mention that in most cases EDC responses, as well as hormones, do not show the typical monotonic dose responses classically used in toxicology studies but rather follow non-monotonic dose responses (NMDR) (16). A first and widely used model in toxicology is the dose addition model (DA). DA assumes that toxicity can be expected if the sum of the concentration of each compound of the mixture is high enough to surpass the threshold of toxicity of the mixture, regardless of the fact that the concentration of each compound is below its own effect threshold (205). A conventional term for threshold is the No Observed Adverse Effect Levels (NOAELs). NOAELs are used to deduce regulatory threshold values applying an uncertainty factor, typically of 100 resulting from a factor of 10 to take into account interspecies extrapolations and another factor of 10 considering intra-species variability (206). Another concept is the principle of Response Addition (RA) used to assess the toxicity of a mixture containing compounds with different modes of action (207). Consequently, models integrating the concepts of DA and RA are needed to investigate complex mixtures of chemicals with both similar and dissimilar modes of action, a so-called “integrated-addition model” (208). Several studies have undertaken a “whole mixture approach.” In this approach a combination of chemicals is considered as a single compound even if specific effects of each component are not investigated (209, 210). While studying complex mixtures is necessary, extrapolating from one mixture to another is difficult knowing that small changes in composition can possibly lead to differences in outcomes (211). An example of application of whole mixture approach is that the *in vivo* identified animal model dose-response relationship of the mixture can determine a reference dose (RfD) associated with an adverse health outcome *in vivo*. A statistical measure of “sufficient similarity” of the RfD can be used to compare the RfD with EDC exposure levels assessed in the human population to generate a “similar mixture risk indicator” in order to identify people at risk. The systematic integration of experimental animal studies and epidemiological studies may improve the scientific understanding of implicated health effects and improve risk assessment of EDCs (212).

### *In vivo* Evidence of Thyroid Disruption

A study exposed rats for 4-days to different TH synthesis inhibitors (pesticides: thiram, pronamide, and mancozeb) and stimulators of T4 liver clearance [polyhalogenated aromatic hydrocarbons (PHAHs): 2 dioxins, 4 dibenzofurans; and 12 PCBs including dioxin-like and non-dioxin like polychlorinated biphenyls (PCBs)], and compared the decrease in T4 serum levels with the three aforementioned models' predictions (response addition, dose addition and integrated addition as explained above). Rats exposed to highest dose of the mixture demonstrated a 45% reduction of serum T4. Results from this study support both the dose- and integrated-addition model as they provided a better prediction than the response-addition model (213). Another study conducted by Wu et al. (214) observed synergistic effects between the organophospate triazophos (TRI) and the neonicotinoid, imidacloprid, when investigating combined toxicities in zebrafish. mRNA levels of *dio1, dio2*, and *tsh* were more affected in joint exposure compared to their individual pesticide exposure, underlining the importance to incorporate joint toxicity studies. Due to specific action of each compound, a mixture of the insecticide IMI in co-exposure with the fungicide mancozeb (MCZ) is frequently used in the field. Therefore, another mixture study used this combination to mimic real-life exposure for both wild animals and human beings. One focus of this study was the assessment of differences in body weight of mice exposed via lactation to low doses of the two pesticides. First, *in silico* molecular docking (MD) was used to predict the probable binding modes of pesticides with THRs (alpha and beta). MD showed both compounds could compete with active TH for TR binding. No effect on body weight was seen with individual exposure whereas low dose exposure to a combination of both pesticides induced significant gain in relative body weight. This increase in weight might be a consequence of TH imbalance as, additionally, significant decreases in THs (both T3 and T4) and increases of TSH in blood plasma were observed in mice exposed to the mixture (202). Another study exposed rats to the four most widely used OP insecticides (dichlorvos, dimethoate, acephate, and phorate) in agriculture in China and identified that these pesticides elicit a joint toxic response. Rats were given the mixture via drinking water for a period of 24 weeks and plasma was analyzed by metabolomics. Levels of iodotyrosine, an early precursor to T4, were decreased, suggesting that exposure to an OP mixture can alter thyroid gland function (215). As there is increasing emphasis on the use of blood-based biomarkers of adverse outcomes in safety assessment, other studies have evaluated targeted metabolite profiles after exposure to different chemical groups (193). Given the “one health” concept, with human health being related to that of biodiversity and the environment, non-target organisms of pesticides are a concern for human well-being. A study on the seasonally breeding finch, *Amandava amandava* demonstrated that when co-exposed to a dithiocarbamate (mancozeb/MCZ) and a neonicotinoid IMI, both thyroid homeostasis and reproductive axis were affected. A 30-day exposure to this mixture via food intake, at concentrations even lower than environmental levels, changed thyroid weight and volume, with decreased T3 and T4 plasma levels (216, 217).

Overall, thyroid-disrupting chemicals are less investigated than estrogen or androgen disrupting compounds, thus very few studies have centered on investigating TH-related endpoints of pesticide mixtures.

## Discussion

### Evidence of PPP Thyroid Disrupting Effects

A significant increase in many neurodevelopmental and neurodegenerative diseases in both adults and children has drawn attention to the potential role of environmental factors, including pesticides (218). Despite the relatively well-known effects of different classes of pesticides on the HPT axis (44, 219), numerous knowledge gaps and mechanistic insights remain between specific thyroid hormone-related endpoints and adverse neurodevelopmental parameters. In this review, after applying a defined methodology, we focused on 45 recent papers that investigated the association of TH disruption and pesticide exposure. A large number of these pesticides are associated with TH axis disruption either in humans or in experimental models.

### Epidemiological Data

In most mother/child cohorts, organochlorine pesticides were associated with either an increase in TSH, a decrease in circulating TH or both (see section Background and Table 1). These pesticides represent the first generation and the majority is banned. However, as they are so persistent, they remain in human fluids and the environment as legacy pesticides, adding to the potential effects of newer pesticides. This second generation pesticides reviewed here, includes, the acetylcholine esterase inhibitors, includes organophosphates (including glyphosate and chlorpyrifos) and carbamates (sections Overview Of The Hypothalamus-Pituitary-Thyroid Axis and Methods). Their main mode of action causes the accumulation of acetylcholine at the synaptic region. Their presence in blood or urine during early development has been associated with thyroid dysfunction (114), increase of hypothyroidism (91, 139, 140), brain function impairment (107, 113), and increase in hormone-dependent cancer (115), which can be related to homeostatic imbalance.

Interestingly, for the last generation of pesticides examined, only Fipronil belonging to the phenylpyrazole family was associated with a TSH decrease (189) in adults or a fT3 decrease level in newborns (169). No other associations were identified with pyrethroids or neonicotinoids despite several disruptive effects on the thyroid axis having been reported in *in vivo* animal studies, along with description of plausible mechanisms *in vitro* (see sections Pyrethroids, Phenylpyrazole, Neonicotinoids). However, the lack of epidemiological studies on TH and neonicotinoid exposure needs to be re-emphasized.

To understand the apparent discrepancy between epidemiological and animal models, some parameters need to be taken into account: (i) a major difference between previous and modern pesticide formulations is the shorter half-life and lower bio-accumulative potential of newer compounds. This implies that active compounds are not necessarily present at the time of sampling. (ii) If absent or below the detection limit, a specific biomarker or metabolite needs to be measured. In all cases, specific analytical methods need to be developed and validated. (iii) We are all exposed to a multitude of chemical and pesticide residues which are not necessarily measurable due to their short half-lives (see following section Underestimated Parameters in Epidemiological Studies). An example, although not a pesticide, is tetrabromobisphenol A(TBBPA) (220). Therefore, combined effects may not be apparent in epidemiological data whereas, in a more systematically controlled experiment, significant effects of a single compound or mixture may be revealed. Moreover, as we are exposed to a multiple of chemicals at a certain time point and geographical location, one has to be extremely cautious about associations made with any single chemical and on a given study population. To date, most data sets rely on basic biomarkers such as TH level measurements and too few studies investigate actual mixture effects.

#### Underestimated Parameters in Epidemiological Studies

(1) Joint exposures
  Few epidemiological studies address mixture effects. This is because current risk assessment focuses on single molecules, with interactions between chemicals rarely taken into consideration. Mixture exposure, representing more realistic scenarios, is unduly underestimated.
  First, legacy pesticides that have been overwhelmingly studied as single compounds are not considered as parts of mixtures we are currently exposed to. Second, synergy, commonly defined as the effect of multiple compounds working in combination that is greater than the expected additive effect of individual chemicals, is rarely considered (221). Further, EDCs can also exert opposing effects on the same axis and cross-talk between different axes has been well-established (222).
  One of the principal aims of epidemiological studies is to clarify complex pictures. Thus, one goal is to determine if an association exists with the lowest occurring parameter. The effects of chemical exposure can be manifold, with pathways induced by banned, persistent or legacy PPP, potentially masking adverse health endpoints through overlapping and, possibly, overlooked mechanisms. Further, absence of association is not necessarily synonymous with chemical inertness.
(2) Long lasting effects and sensitization
  Pesticides may not exert an obvious adverse effect at the moment of exposure, but effects may be delayed or one exposure may sensitize the organism to a later occurrence, incurring a fitness cost, which might only be seen when a second stressor appears or later in life.
  Different scenarios can be envisaged. The following examples may not refer specifically to pesticides but serve to illustrate the point. First, alterations of the immune system can be implicated. Recent studies revealed modifications in immune responses following exposure to a mixture of molecules used for fracking and found in drinking water. This has been shown in mouse and amphibian models following developmental exposure to a “fracking” mixture. Prior exposure resulted in increased mortality when the animals had to cope with an otherwise benign viral infection (223, 224). These altered immune responses echo epidemiological studies such as in the Faeroe islands reported by Grandjean and Andersen (225). They showed that children exposed to perfluorinated compounds displayed reduced antibody responses to a standard vaccination protocol.
  Second, other environmental factors may act as secondary stressors, e.g., another xenobiotic affecting the same or another essential physiological pathway or the same xenobiotic at a different time-point, to which one can add climate change and/or reduced insect populations during bird migration. All of these parameters need to be borne in mind when attempting to understand the more far reaching effects of pesticides on non-target species, including humans.

#### Evidence for *in vivo* and *in vitro* Mechanisms of Action

One could summarize that organochlorine and organophosphates were designed to stably block enzymes used in the biochemical processes of the pest targeted. Most novel pesticides were designed to address problems of accumulation (most pronounced in compounds classified in the Stockholm convention as persistent organic pollutants (POPs), pesticide-induced pest resistance and high mammalian toxicity at low doses. In theory, these second generation molecules can be used at higher concentrations, as well as being more specific and biodegradable. However, their impact on non-target organisms are often neglected and only toxicity assays were (and still are) required before release on the market. As illustrated by the OP chlorpyrifos, pesticides may have cryptic effects at sub-lethal concentrations (119, 120, 226).

**Figure 2:**
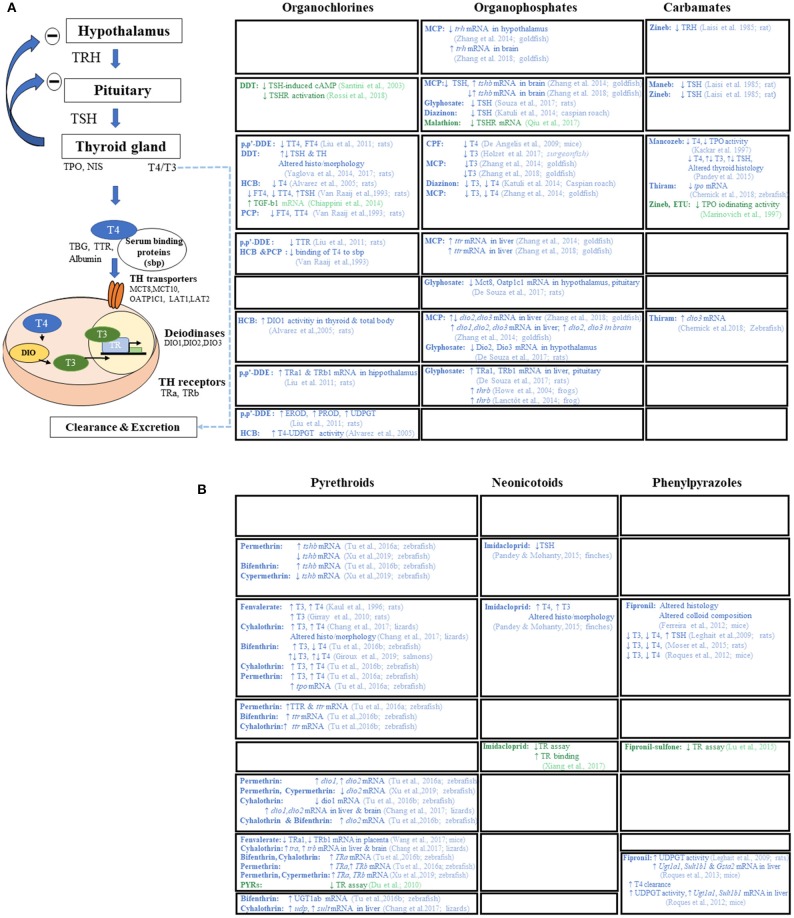
Mode(s) of action of pesticides reported by *in vivo* (in blue) and *in vitro* (in green) articles. **(A)** Mode(s) of action of 1st and 2nd generation of pesticides (organocholorines, organophosphates, and carbamates). **(B)** Mode(s) of action of newer pesticide families (pyrethroids, neonicotinoids, and phenylpyrazoles). TRH, thyrotropin-releasing hormone; TSH, thyroid stimulating hormone; T4, thyroxine; T3, triiodothyronine; TPO, thyroperoxidase; NIS, sodium/iodide symporter; TBG, thyroxin binding globulin; TTR, transthyretin; MCT, monocarboxylate transporters (mct8, mct10); OATP1C1, organic anion-transporting polypeptide 1c1; LAT, large neutral amino acid transporter (lat1 and lat2); DIO, deiodinases (dio1, dio2, dio3); TR, thyroid hormone receptor (thra, thrb); UGT1ab, UDP-glucuronyltransferase 1ab; UDPGT, UDP-glucuronyltransferase; SULT, sulfotransferase; EROD, ethoxy-resorufin-O-deethylase; PROD, pentoxy-resorufin-O-deethylase; GSTA2, glutathione S-transferase alpha 2; cAMP, Cyclic adenosine monophosphate; TGF-b1, transforming growth factor b1; DDT, dichlorodiphenyl trichloroethane; p,p′DDE, p,p′-dichlorodiphenyl dichloroethylene; HCB, hexachlorobenzene; PCP, pentachlorophenol; MCP, monocrotophos; CPF, chlorpyrifos; ETU, ethylenethiourea.

All classes of pesticides have been associated with disruptive effects of the HPT axis at various levels determined by *in vitro* and/or *in vivo* studies (see Figure 2). Strikingly, none of these actions are currently included in assays required before a PPP is marketed. One must note that detection of disruption at a given level is insufficient to identify adverse action of all pesticides (even within different pesticide classes). While the abnormality might only be transient, persistence may be due to underlying thyroid dysfunction. More, as shown for OCPs, some chemicals may have opposite effects on circulating TH. Interestingly in Freire et al. (75), an endosulfan metabolite was associated with a decrease in TSH whereas other OCPs were associated with an increase in TSH. While *in vivo* studies suggest a mechanism based on increased clearance by hepatic activation of phase II enzymes, *in vitro* studies suggest direct action on TH synthesis at the thyroid gland level highlighting the complementarity of approaches.

Both the EFSA scientific report (44) on cumulative assessment groups of pesticides and annex A of the guidance document for the identification of EDCs, state that all molecules acting on the liver, increasing metabolism or modifying deiodinase 1 activity with consequent TH levels modifications, can be considered as TH axis-disrupting compounds. This feature represents a large proportion of pesticides and is a significant step toward identifying chemicals affecting the thyroid axis. Hence, pesticides may also exert effects beyond TH levels and the thyroid gland, with displacement from distributor protein and increased liver metabolism (e.g., fipronil-sulfone, see Figure 2 for a detailed review on level of actions of different pesticides on TH axis).

### A Need for Improved Testing Strategy, Risk Assessment, and Updated Legislation

As Milner and Boyd already discussed in 2017, “pesticidovigilance” is something that needs to be developed. One of the possible strategies is to transpose what is currently carried out in pharmacovigilance for drugs, to PPPs or other classes of chemicals. This step would be beneficial for human, animal and environmental health (227).

#### Improved Testing

The vast majority of the available validated assays by EPA or OECD were developed from former reproductive or toxicological assays enriched with endocrine related endpoints, i.e., TH levels measurements and thyroid gland histology. Therefore, these assays were not specifically designed to detect endocrine sensitive or specific endpoints. The central nervous system is one of the major targets of TH and therefore, TH-axis disruptors. Consequently, we sorely need better brain endpoints for potential effects of pesticide exposure taking into account the multiple levels of potential interference. In the brain, cell proliferation, neural stem cell differentiation, neuronal migration, synaptogenesis or myelination can all be influenced by TH availability. For example, in neonatal rats, prenatal exposure to the anti-thyroid compound PTU at low doses induces failure in neuronal migration—an event more sensitive than circulating TH levels (228). Given this overall lack of sensitivity, the EU commission is currently funding three large-scale projects (ATHENA, ERGO, and SCREENED) to develop innovative and specific endpoints for TH axis disruption, specifically with a focus on brain endpoints.

#### Risk Assessment

The report by Demeneix and Slama for the EU parliament in early 2019 highlighted the steps necessary for efficient risk management in terms of protecting Health and environment: (1) a hazard definition followed by the (2) availability of a guidance document to allow reproducible application of the definition (3) specific validated assays (4) test requirements and (5) integration of the results in a risk management strategy (229). Only steps 1, 2 and 5 are fulfilled for PPPs. Current risk assessment cannot integrate specific endpoints and mixture effects, though innovative approaches have been developed to overcome these limitations for compounds other than pesticides (230).

#### Legislation

A definition for EDCs and criteria relative to PPP and biocides was adopted in 2018 in the EU. A guidance document on identification of endocrine disruptors for PPP and biocides was approved in June 2018 (231). Nevertheless, major gaps still exist in order to appropriately regulate PPP, essentially attributable to the current lack of specific endpoints. Recognition of the effects of mixtures is making progress as highlighted by EFSA submitting a document for public consultation on the establishment of cumulative assessment groups of pesticides for their effects on thyroid signaling (232).

## Conclusion

The recent data reviewed here show that in humans, TSH, T3, and T4 measurements are still the most common endpoints evalutated in order to address thyroid disruption and pesticide exposure. Epidemiological data reveal that legacy pesticides (Organochlorine, organophosphate, carbamates) are more often associated with thyroid axis disruption than modern compounds (pyrethroids, neoinicotinoids, and phenylpyrazoles). However, experimental work demonstrates, with both *in vivo* and *in vitro* evidence, that each pesticide class disrupts thyroid homeostasis at different levels. Therefore, one should consider systematizing TH measurements, determining more sensitive brain endpoints for assessing TH disruption and carrying out more detailed longitudinal studies in epidemiology. As the OECD and the EU Commission have underlined, revisiting thyroid axis-disrupting chemicals is a major research priority.

## Take Home messages

- Multiple arguments link environmental causes to the increased incidence of neurodevelopmental disease that many authors have suggested could be related to altered TH signaling.
- Experimental studies show that different classes of pesticides can act at multiple levels on TH signaling.
- Most often, data from epidemiological studies measure levels of TSH and/or TH in blood. These endpoints may not be sensitive enough to detect the small changes that might occur with newer pesticide formulations.
- Hence, there is a need for novel TH and brain specific endpoints that are more sensitive to variations in TH.
- A final point is the need to take the mixture effects (of pesticides and other EDCs) into consideration in risk assessment and regulatory purposes.

## Author Contributions

ML and J-BF conceptualized and designed the study and conducted pre-screening on abstracts. ML, J-BF, and SC identified the Mesh terms. ML and SC enriched the Mesh Terms and reviewed all the articles and interpreted the data. ML drafted the figure. ML, SC, BD, and J-BF edited the manuscript. All authors read and approved the manuscript.

### Conflict of Interest

BD is co-founder of Watchfrog but receives no financial compensation. The remaining authors declare that the research was conducted in the absence of any commercial or financial relationships that could be construed as a potential conflict of interest.

## Footnotes

^1^https://ec.europa.eu/food/plant/pesticides_en

^2^dichlorodiphenyl trichloroethane (DDT).

^3^p,p'-dichlorodiphenyl dichloroethylene (p,p'-DDE).

^4^World Health Organization.

^5^http://ec.europa.eu/food/plant/pesticides/eu-pesticides-database

^6^http://mesh.med.yale.edu/

^7^National Health and Nutrition Examination Survey.

^8^https://www.bbc.com/news/world-europe-40878381

## Supplementary Material

Table S1 All articles (223) classified by pesticide class with justification for selection or omission.
